# Effect of Test Parameters on the Friction Behaviour of Anodized Aluminium Alloy

**DOI:** 10.1155/2014/795745

**Published:** 2014-10-29

**Authors:** M. Guezmil, W. Bensalah, A. Khalladi, K. Elleuch, M. De-Petris Wery, H. F. Ayedi

**Affiliations:** ^1^Laboratoire de Génie des Matériaux et Environnement (LGME), ENIS, Université de Sfax, B.P. 1173-3038, Tunisia; ^2^IUT Mesures Physiques d'Orsay, Université Paris Sud, Plateau du Moulon, 91400 Orsay, France

## Abstract

The tribological behaviour of anodic oxide layer formed on Al5754, used in automotive applications, was investigated against test parameters. The friction coefficient under different normal loads, sliding speeds, and oxide thicknesses was studied using a pin on disc tribometer. Results show that the increase of load and sliding speed increase the friction coefficient. The rise of contact pressure and temperature seems to cause changes in wear mechanism. Glow-discharge optical emission spectroscopy (GDOES) was used to investigate the chemical composition of the oxide layer. Morphology and composition of the wear tracks were analyzed by scanning electron microscopy (SEM) and energy dispersive X-ray spectroscopy (EDS). On the basis of these characterization techniques, a wear mechanism was proposed. The observed mechanical properties can be related to the morphology and the chemical composition of the layer.

## 1. Introduction

Anodic oxide layers are of increasing interest for many engineering applications (automotive, shipbuilding, and construction industries) due to their high hardness and high resistance to thermal and corrosive loadings combined with relatively low densities [1–5].

The anodic oxide layer has porous structure and mainly consists of amorphous Al_2_O_3_ [6–8].

Owing to this porosity, anodic oxide layer is very beneficial for tribological application as they can be used as a reservoir for lubricants to form self-lubricating structures [9–11]. Many researchers have demonstrated the enhancement of friction and wear performance of the anodized aluminum by using plasma electrolytic oxidation and/or by modifying the anodic structure [12, 13].

However, we think that the tribological behavior of anodic oxide layer itself needs to be more investigated to be well utilized as self-lubricating structures.

Mezlini et al. [14] have investigated the effect of sulfuric anodizing (SA) on the scratch damage of the 5xxx aluminum alloy used in transport application. For scratch test, they have found that SA treatment decreases abrasive wear resistance despite the increase of the anodized layer hardness.

Kim et al. [7] have studied the tribological behavior of nanoporous structure films with various pore sizes in sliding contact with steel ball, varying the normal load from 1 mN to 1 N. They have shown the decrease of the friction coefficient with the increase of load. They attributed this fact to the formation of smooth and thick tribolayer at high loads.

Lee et al. [15] have used ball-on-disk wear tests to study the tribological behavior of porous anodic alumina. They have concluded that water stored inside the pore has great effect when it is released during sliding by elastic-plastic deformation.

The main focus of this paper is to study the effect of normal load, sliding speed, and oxide thickness on the tribological behaviour of anodic oxide layer formed on Al5754 in sulphuric acid bath. Moreover, worn surfaces were analyzed using scanning electron microscopy (SEM) coupled with energy-dispersive spectroscopy (EDS). Glow-discharge optical emission spectroscopy (GDOES) was used to find out the chemical composition of anodic layers.

## 2. Experimental

### 2.1. Materials and Methods

In this study, the 5754H111 aluminium alloy was used. Samples with dimensions of 100 × 25 × 3 mm^3^ were mechanically polished to P1000 grade paper to obtain smooth surfaces followed by (i) chemical polishing in a 15/85% (v/v) mixture of concentrated phosphonitric acids (HNO_3_ + H_3_PO_4_) at 85°C for 2 min, (ii) etching in 1 M sodium hydroxide NaOH solution at room temperature for 1 min, and (iii) chemical pickling in 30% (v/v) HNO_3_ solution at room temperature for 30 s. Water rinsing was used after each step. Afterwards, samples were anodized in vigorously stirred 160 g L^−1^ sulphuric acid bath maintained at 15 ± 0.1°C under 2 A·dm^−2^. The anodizing duration was chosen so that to obtain oxide layer with 30 *μ*m of thickness measured using ELCOMETER 355 Top Thickness Gauge equipped with eddy current probe. It is to mention that the used cathodes were also aluminium sheets.

### 2.2. Testing Methods

#### 2.2.1. Friction Test

Friction tests were carried out in dry condition using a pin-on-disc tribometer. Anodized samples with dimensions of 20 × 20 × 3 mm^3^ were brought into contact with 100C6 steel ball with a diameter of 6 mm. All tests were performed at the same sliding speed of 100 tr/min (0.052 m/s). Friction tests were performed in ambient air (25–27°C) at relative humidity (RH) of 35–45%. During tests, the variation of the friction coefficient versus time was recorded.

#### 2.2.2. Surface Morphology

The morphology of the oxide layer was studied from the top side of the layer using a scanning electron microscope SEM-FEG (Jeol JSM-6400F). Atomic force microscopy (AFM) characterization, using model digital instrument-nanoscope probe II (contact mode), was realized to examine the roughness of the anodized surfaces. Surface topography was recorded over scanned areas of 5 *μ*m × 5 *μ*m.

Surface profiles, using Surtronic 25 profiler from Taylor Hobson, was used to examine the surface topography of the different oxide layers.

The wear tracks were studied using a LEICA optical microscope and a TESCAN VEGA II scanning electron microscope (SEM) coupled with an energy-dispersive X-ray spectroscopy (EDS) for chemical analysis.

#### 2.2.3. Glow-Discharge Optical Emission Spectroscopy (GDOES)

The distribution of chemical species in the anodic oxide layer was determined by depth profiling using a Jobin Yvon GD Profiler instrument equipped with a 4 mm diameter anode and operating at a pressure of 800 Pa and a power of 600 W in an argon atmosphere. The relevant wave lengths (nm) were as follows: Al, 396.15; O, 130.22; S, 181.73; and C, 156.14. The sputtering layer was 6 *μ*m thick.

## 3. Results and Discussion

### 3.1. Friction Coefficient of the Al5754 Substrate

**Figure 1:**
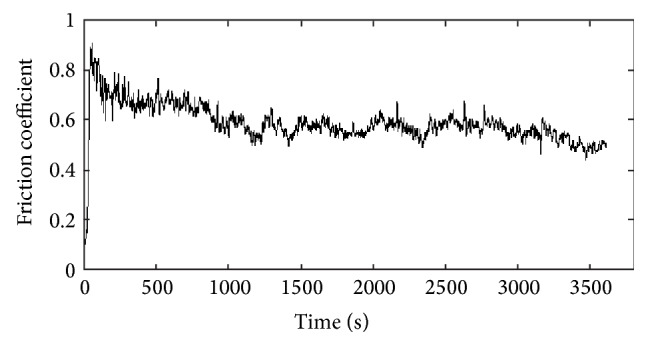
Friction coefficient of Al5754 as a function of sliding time.

Before studying different anodic oxide layers, the friction behaviour of the aluminium substrate was investigated (Table 1). The variation of the friction coefficient versus time is shown in Figure 1. As can be seen, this plot reveals three zones connected to three regimes: friction first increases, then increases, and finally achieves a steady state value for the remainder of the sliding distance. The changes of the friction coefficient values can be associated with changes in the wear morphology and the extent of oxidation. These results are in accordance with those of Kim et al. [16]. In fact, they demonstrated the role of oxygen on the wear morphology evolution. Besides, Yerokhin et al. [17] relate this finding to the transition from the wear mechanism of the couple steel/aluminium (ductile) to that of steel/oxide film (brittle) formed by oxidation which decreases the friction coefficient.

**Figure 2:**
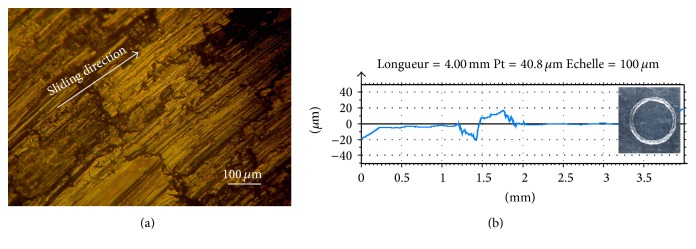
(a) Optical microscopy of worn surface of the aluminium substrate. *F* = 1 N, *V* = 100 tr/min; (b) wear track profile.

Figure 2(a) shows the wear track after the friction test. The surface roughness has increased. The worn surface was smooth, distinct parallel and continuous grooves with plastic deformation can be observed. This figure shows that severe adhesion took place.  Figure 2(b) shows the profilometric measurement undertaken on the wear track. The obtained track depth and width were 20 *μ*m and 0.75 mm, respectively.

### 3.2. Effect of the Oxide Layer Thickness on the Friction Coefficient Response

**Figure 3:**
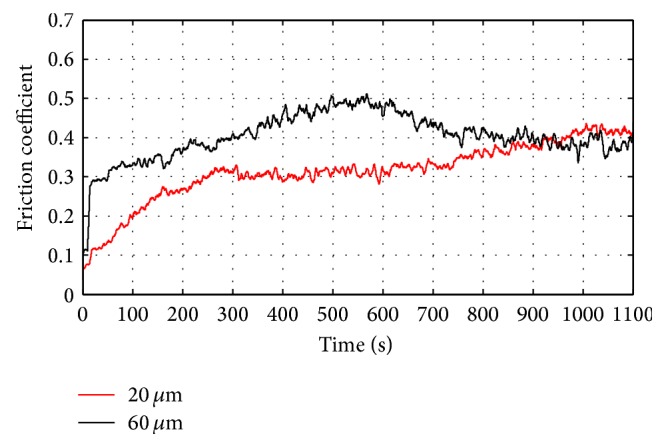
Effect of the oxide layer thickness on the friction coefficient response. *F* = 1 N, *V* = 100 tr/min.

Figure 3 depicts the friction coefficient evolution versus time for anodic oxide layer with two thicknesses 20 and 60 *μ*m. During the 10 first minutes of the test, the friction coefficient shows an increase for thickness of 60 *μ*m, and then it reaches, for both layers, similar value. In fact, thick coatings have long and deep pores with more open structures because of the long residence time in the anodizing bath. These coatings are more subjected to major collapse under the contact pressure. This finding was equally observed by Fratila-Apachitei et al. [18] when they measured Vickers microhardness of anodic oxide layer with different thicknesses. In fact, they have found that the hardness decreases when going from oxide/substrate to oxide/electrolyte interface due to the chemical dissolution reactions at the outer interface. Yerokhin et al. [17] have studied the effect of the anodic oxide layer thickness on the friction response. They demonstrated that coatings with low thicknesses are more effective in terms of friction, scratch, and impact resistance.

### 3.3. Effect of the Normal Load on the Friction Coefficient Response

The anodization layer is porous and brittle; applying significant normal load during a friction test can lead to its failure. In fact, some authors like Kim et al. [7] have used very low loads (1 mN–1 N) when working on the friction behavior of anodic oxide films on aluminum. These authors showed that a load of 1 N is considered high and can lead to severe plastic deformation of compacted debris and the formation of smooth and thick tribolayer. In this context, we have chosen to run friction tests with loads between 1 and 3 N in order to avoid over contact stress and losing information on the contact between the mating materials.

**Figure 4:**
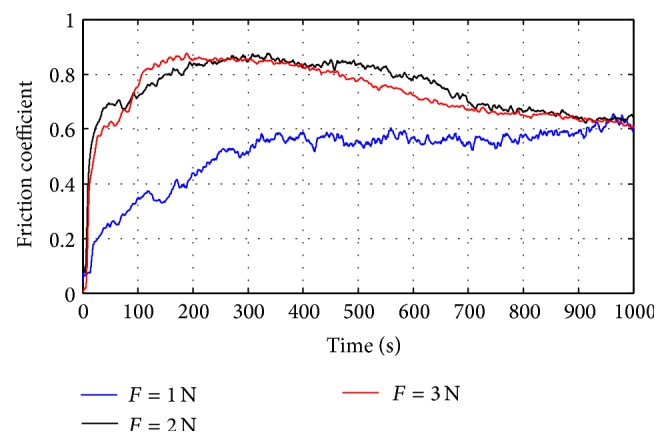
Effect of the normal load on the friction coefficient response: *V* = 100 tr/min.

Figure 4 shows the variation of the friction coefficient of the anodic oxide layer versus time, depending on the normal load. As can be seen, the friction curves show a transition period during the first cycles in which the coefficient is high. Then, the friction coefficient decreases to a stable value: the steady state. This figure shows that under the same sliding speed, the friction coefficient increased with the load and the greater the load was, the longer the time would be from the beginning of stabilization.

**Figure 5:**
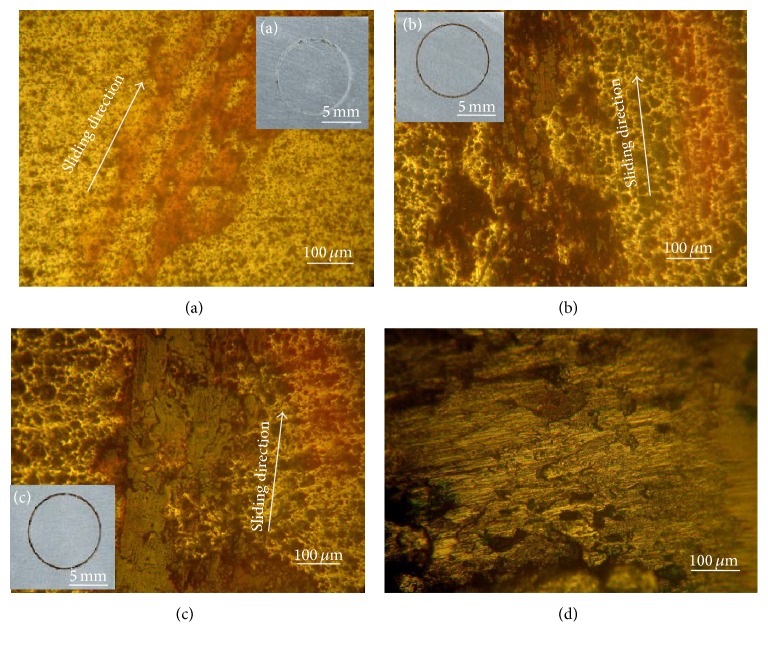
Optical microscopy images of the wear tracks as a function of normal load: (a) *F* = 1 N; (b) *F* = 2 N; (c) *F* = 3 N; and (d) surface of the stainless steel ball.

Figure 5 shows an overview of wear tracks of worn oxide layers under different loads. The analysis of this figure shows that, beyond a load of 2 N (Figures 5(b) and 5(c)), the rapid formation of black layer at the wear contact was observed. This phenomenon was usually accompanied by the increase in the friction coefficient at the beginning of the test. For a load of 1 N (Figure 5(a)), this fact occurs in a delayed way. This finding was observed also by Wang et al. [19] and Rapheal et al. [20]. Indeed, they showed that the increase of the load (5N) allows the rapid establishment of transfer phenomena. For loads less than 2 N, the stationary state is delayed.

Figures 5(a)–5(c) show the presence of smooth and thick layers formed by a combined action of tribochemical reactions of oxidation and transfer in the contact by severe plastic deformation of crushed debris.

This phenomenon was observed by Kim et al. [7]. These authors have shown, by chemical analysis using EDS technique, that the studied layer is composed of iron and chromium, the elements of the 440C stainless steel ball and of Al, S, O, and C components of the anodizing layer. Indeed, the observation by optical microscopy of the contact area at the steel ball shows a remarkable degradation (Figure 5(d)).

In the beginning of the friction test, owing to the strong adhesion between the steel ball and the oxide layer, the metal of the ball was transferred to the coating surface. After some time had elapsed, the oxide surface was entirely covered by a layer of transferring metal that could have formed chemical compounds with the oxide layer. It can be seen from the friction coefficient curves that the friction coefficient increases initially with increasing sliding distance, this being called the running-in period, and then reached an equilibrium with a more stable value, which may be caused by the transfer film formed on the sliding surface [21].

It remains to note that the increase of the load causes an increase in temperature at the frictional contact. The heat generated accelerates the oxidation phenomena and transfer. This phenomenon was also observed by Kim et al. [7].

The increase in temperature at the contact may be the result of the transferred film (mostly FeO, judging by the rust color) which would be more easily adhered to the anodic oxide layer under higher loads than that under lower loads. Also, the wear condition would become steel-to-steel wear and the transferred film would act to reduce the friction coefficient between the sliding surfaces.

More heat generated at the wear interface causes higher oxidation of the iron surface and thus an oxide film might form to provide a degree of lubricant and protect the steel ball from excessive wear [21].

### 3.4. Effect of the Sliding Speed on the Friction Coefficient Response

**Figure 6:**
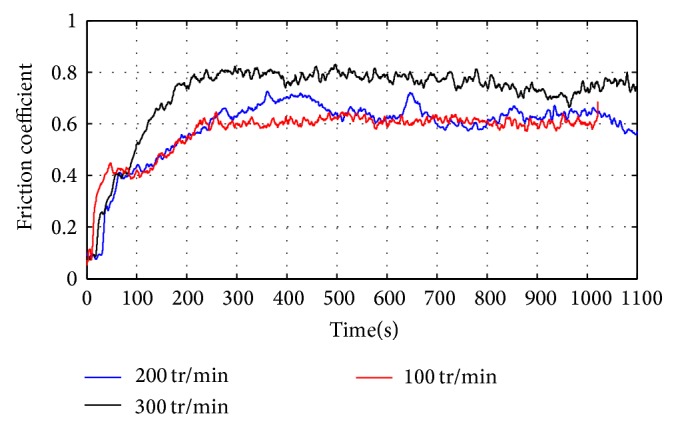
Effect of the sliding speed on the friction coefficient. Normal load *F* = 1 N.

Figure 6 shows the variation of the friction coefficient of anodic oxide layer as a function of the sliding speed under normal load of 1 N. The examination of this figure shows that the friction coefficient seems to increase with sliding speed.

**Figure 7:**
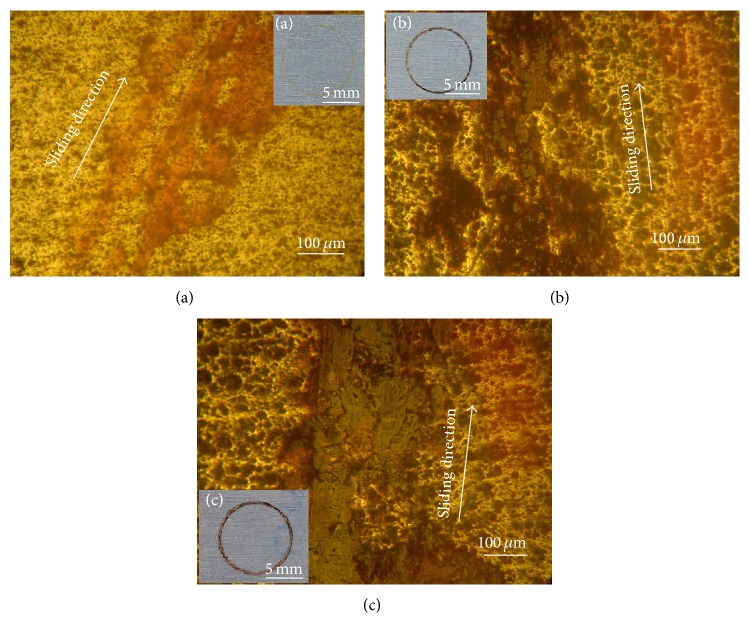
Optical microscopy images of the wear tracks as a function of sliding speed: (a) *V* = 100 tr/min; (b) *V* = 200 tr/min; and (c) *V* = 300 tr/min. Normal load *F* = 1 N.

An overview of wear tracks generated on tested samples is shown in Figure 7. The formation of the oxidation layer is even more pronounced when the speed increases. These findings were also highlighted by Kim et al. [7]. These authors attributed the change in friction behaviour to the contact temperature. Indeed they showed that high speeds generate more heat at contact, thus facilitating the oxidation and transfer phenomena and the formation of tribolayers (Figure 7).

### 3.5. Wear Mechanism

**Figure 8:**
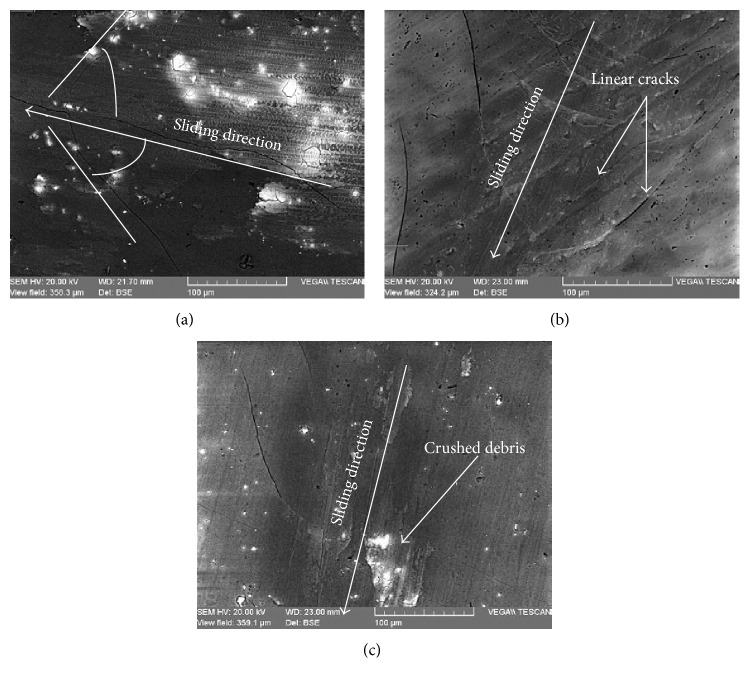
SEM images of wear tracks on tested anodic oxide layers (a): *V* = 100 tr/min, normal load *F* = 1 N; (b) *V* = 100 tr/min, normal load *F* = 3 N; and (c) *V* = 300 tr/min, normal load *F* = 1 N.

Figure 8 demonstrates the formation of smooth layers at the contact surface. These layers are created by the combined influence of tribochemical reaction and material transfer. The observed smoothness suggested the generation of severe plastic deformation of compressed debris (Figure 8(c)).

Figure 8 shows the existence of surface polishing, microcracking, and detachment of wear particle. Microcracks were propagated outside the contact zone on both sides of the sliding direction following the frontward movement of the steel ball. All cracks have identical angles with regards to the sliding direction (Figure 8(a)). It means that, during sliding, the cracks are together formed on both sliding sides along the wear scars.

It seems that microcracks are produced due to the presence of shear stresses during the friction test. Hence, the pores of anodic oxide layer were deformed by extrusion pressure and covered with abrasive particles. These coatings do not exhibit any deeper scratches, but microcracking assisted flaking is found to be the predominant wear mechanism [22, 23].

**Figure 9:**
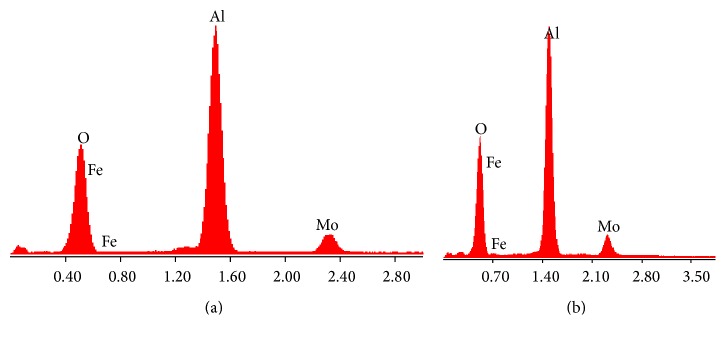
Typical EDS analysis of wear debris and worn surfaces of anodic oxide layers.

Figure 9 shows typical EDS spectrum carried out on the tribolayers and wear particles formed after friction test. This figure reveals that these tribolayers were composed of Fe and Mo, transferred from the ball, and Al and O, the main chemical elements of the oxide layer. It explains that active tribochemical reaction and material transfer occurred between the steel ball and the oxide layer.

**Figure 10:**
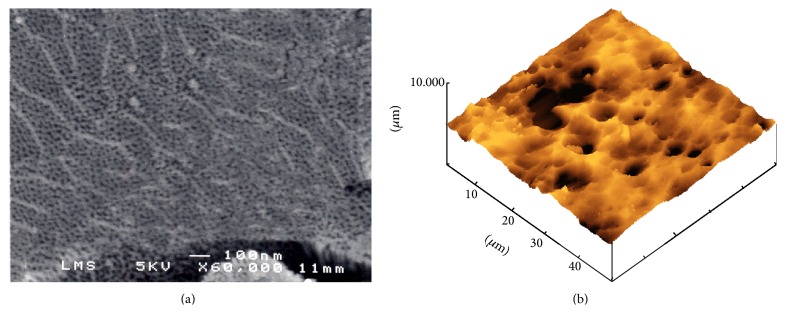
Typical (a) SEM and (b) AFM images of anodized layer.

Here it is to mention that the tribological behavior goes with the evolution of anodic layer porosity [6]. The anodized layer presents open porosities, cavities, and some local perturbation attributed to the elaboration conditions.  Figure 10 shows typical SEM and AFM images of the anodized layer. As can be seen, pores were distributed macroscopically in a random fashion. They had a uniform size and were arranged in a honeycomb hexagonal structure.

The topography obtained by AFM shows the presence of cavities and open porosities. From a geometrical point of view, the porosity of the layer can affect its friction behaviour. In fact, the movement of the steel ball will not be the same on large and small pore diameters and/or on flat or disturbed morphology.

The alloying element seems to affect the friction behaviour of the anodic layers formed on Al5754. In fact, the formed layer on Al5754 contains, with Al_2_O_3_, the magnesium oxide (MgO) [24]. It is well referred in the literature [4] that, during the anodizing of the Al-Mg alloys, direct oxidation of Mg takes place and the MgO will be formed with the alumina film. Moreover, the Mg^+2^ ions have outward mobility about 1.5 times greater than that of Al^3+^ ions and associated with single metal-oxygen bond strength. These facts will result in a uniform distribution of MgO oxide through the alumina layer. In the same context, Zhou et al. [4] established that Mg^2+^ ions are ejected more rapidly to the oxide/electrolyte interface which permits a uniform distribution of MgO through the oxide layer.

**Figure 11:**
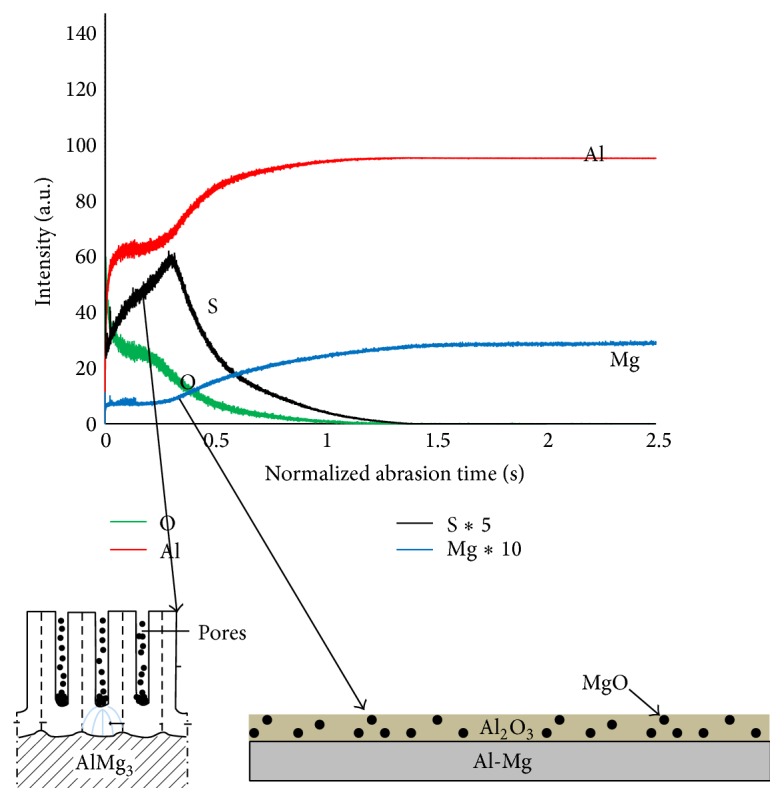
GDOES of typical anodic oxide layer formed on Al5754.

Chemical analysis of the anodic oxide layer was conducted using GDOES (Figure 11). Figure 11 shows the presence of magnesium, aluminium, oxygen, and sulphur. The presence of Mg, Al, and O can be attributed to the MgO and Al_2_O_3_ formation [24]. These results are in accordance with those of Theohari and Kontogeorgou [25]. In fact they have conducted EDS analysis on thick anodized layers on AA5052 formed in 175 g/L H_2_SO_4_ at 15 V. The obtained results reveal that the mean amount of the magnesium element inside the film is about 50–70% less than that in the bulk alloy.

The existence of sulphur can be explained by the inward migration of sulphate anions through the pores of the coating [26]. However, it is possible that sulphur species incorporated from the sulphuric acid bath into the oxide during anodizing grants more compactness and/or lubricity to the anodized layer. Accordingly, it is possible to relate, to some level, the friction behaviour of anodic oxide layer to the anion-contamination.

## 4. Conclusions

In this work, friction tests of anodic oxide layer elaborated on Al5754 substrate against 100C6 ball were conducted. The influence of test parameters on the friction coefficient was highlighted. The results of the present investigation can be summarized as follows:

1. Anodic oxide layers with low thicknesses seem to be more effective in terms of friction.
2. The friction coefficient increases with load and sliding speed. The increase of these two parameters allows the rapid establishment of transfer phenomena. Their increase causes an increase in temperature at the frictional contact. The heat generated accelerates the oxidation phenomena and transfer.
3. Microcracking assisted flaking is found to be the predominant wear mechanism of the anodic oxide layer.
4. GDOES shows the presence of Mg and Al elements suggesting the oxidation of the substrate and the formation of Mg^2+^ and Al^3+^ ions. The presence of oxygen at the substrate/electrolyte interface will conduct to the MgO and Al_2_O_3_ formation.
5. SEM and EDS analysis of tribolayers and wear debris reveal the existence of Fe and Mo, transferred from the ball, and Al and O, the main chemical elements of the oxide layer. This suggests the formation of tribochemical reactions and material transfer between the mating materials.

## Figures and Tables

**Table 1:** Chemical composition of Al5754 aluminium substrate.

Element	Si	Fe	Cu	Mn	Mg	Cr	Zn	Ti	Al
Weight (%)	<0.40	<0.40	<0.10	<0.50	2.6–3.6	<0.30	<0.20	<0.15	Rest

## References

[1] Young L. (1961). *Anodic Oxide Films*.

[2] Wernick S., Pinner R., Sheasby P. (1987). *The Surface Treatment of Aluminium and Its Alloys*.

[3] Bensalah W., Feki M., Wery M., Ayedi H. F. (2011). Chemical dissolution resistance of anodic oxide layers formed on aluminum. *Transactions of Nonferrous Metals Society of China*.

[4] Zhou X., Thompson G. E., Skeldon P., Wood G. C., Shimizu K., Habazaki H. (1999). Film formation and detachment during anodizing of Al-Mg alloys. *Corrosion Science*.

[5] Sheasby P. G., Pinner B. (2001). *The Surface Treatment and Finishing of Aluminium and Its Alloys*.

[6] Aerts T., Dimogerontakis T., De Graeve I., Fransaer J., Terryn H. (2007). Influence of the anodizing temperature on the porosity and the mechanical properties of the porous anodic oxide film. *Surface and Coatings Technology*.

[7] Kim H., Kim D., Lee W., Cho S. J., Hahn J., Ahn H. (2010). Tribological properties of nanoporous anodic aluminum oxide film. *Surface and Coatings Technology*.

[8] Keller F., Hunter M. S., Robinson D. L. (1953). Structural features of oxide coatings on aluminum. *Journal of Electrochemical Society*.

[9] O'Sullivan J. P., Wood G. C. (1970). The morphology and mechanism of formation of porous anodic films on aluminium. *Proceedings of Royal Society of London*.

[10] Maejima M., Saruwatari K., Takaya M. (2000). Friction behaviour of anodic oxide film on aluminum impregnated with molybdenum sulfide compounds. *Surface & Coatings Technology*.

[11] Hu N., Ge S., Fang L. (2011). Tribological properties of nano-porous anodic aluminum oxide template. *Journal of Central South University of Technology*.

[12] Alba-Elías F., Sainz-García E., González-Marcos A., Ordieres-Meré J. (2013). Tribological behavior of plasma-polymerized aminopropyltriethoxysilane films deposited on thermoplastic elastomers substrates. *Thin Solid Films*.

[13] Ma C., Zhang M., Yuan Y., Jing X., Bai X. (2012). Tribological behavior of plasma electrolytic oxidation coatings on the surface of Mg8Li1Al alloy. *Tribology International*.

[14] Mezlini S., Elleuch K., Kapsa Ph. (2007). The effect of sulphuric anodization of aluminium alloys on contact problems. *Surface & Coatings Technology*.

[15] Lee G., Choi J. H., Choi Y. C., Bu S. D., Lee Y. (2011). Tribological effects of pores on an anodized Al alloy surface as lubricant reservoir. *Current Applied Physics*.

[16] Kim H.-J., Emge A., Karthikeyan S., Rigney D. A. (2005). Effects of tribooxidation on sliding behavior of aluminum. *Wear*.

[17] Yerokhin A. L., Nie X., Leyland A., Matthews A., Dowey S. J. (1999). Plasma electrolysis for surface engineering. *Surface and Coatings Technology*.

[18] Fratila-Apachitei L. E., Duszczyk J., Katgerman L. (2003). AlSi(Cu) anodic oxide layers formed in H_2_SO_4_ at low temperature using different current waveforms. *Surface and Coatings Technology*.

[19] Wang Y. M., Jiang B. L., Guo L. X., Lei T. Q. (2006). Tribological behavior of microarc oxidation coatings formed on titanium alloys against steel in dry and solid lubrication sliding. *Applied Surface Science*.

[20] Rapheal G., Kumar S., Blawert C., Dahotre N. B. (2011). Wear behavior of plasma electrolytic oxidation (PEO) and hybrid coatings of PEO and laser on MRI 230D magnesium alloy. *Wear*.

[21] Ming-Chang J., Li-Yung Y. (1993). Environmental effects on wear behaviour of alumina. *Wear*.

[22] Jiang Y., Zhang Y., Bao Y., Yang K. (2011). Sliding wear behaviour of plasma electrolytic oxidation coating on pure aluminium. *Wear*.

[23] Sabatini G., Ceschini L., Martini C., Williams J. A., Hutchings I. M. (2010). Improving sliding and abrasive wear behaviour of cast A356 and wrought AA7075 aluminium alloys by plasma electrolytic oxidation. *Materials and Design*.

[24] Alisch G., Nickel D., Lampke T. (2011). Simultaneous plasma-electrolytic anodic oxidation (PAO) of Al-Mg compounds. *Surface & Coatings Technology*.

[25] Theohari S., Kontogeorgou C. (2013). Effect of temperature on the anodizing process of aluminum alloy AA 5052. *Applied Surface Science*.

[26] Shimizu K., Habazaki H., Skeldon P., Thompson G. E., Wood G. C. (2000). Migration of sulphate ions in anodic alumina. *Electrochimica Acta*.

